# Detoxification, Apoptosis, and Immune Transcriptomic Responses of the Gill Tissue of Bay Scallop Following Exposure to the Algicide Thiazolidinedione 49

**DOI:** 10.3390/biom9080310

**Published:** 2019-07-27

**Authors:** Cheng Chi, Sib Sankar Giri, Jin Woo Jun, Hyoun Joong Kim, Saekil Yun, Sang Wha Kim, Jeong Woo Kang, Se Chang Park

**Affiliations:** 1 Key Laboratory of Aquatic Nutrition and Feed Science of Jiangsu Province, National Experimental Teaching Center for Animal Science, College of Animal Science and Technology, Nanjing Agricultural University, Weigang Road 1, Nanjing 210095, China; 2 Laboratory of Aquatic Biomedicine, College of Veterinary Medicine and Research Institute for Veterinary Science, Seoul National University, Seoul 08826, Korea; 3 Department of Aquaculture, Korea National College of Agriculture and Fisheries, Jeonju 54874, Korea

**Keywords:** algicide, Thiazolidinedione 49, bay scallop, transcriptomic analysis, apoptosis, detoxification, immunity

## Abstract

Thiazolidinedione 49 (TD_49_), a newly synthesized algicide, shows strong toxicity at low concentrations of 0.1–2.0 μM. However, its potential effects on non-target species at the transcript level were not investigated. Differentially expressed genes (DEGs) in the gills of the bay scallop, *Argopecten irradians*, were accessed after treatment with 0.68 μM TD_49_ for up to 48 h. Following exposure, it was observed that 5214 genes were upregulated and 3497 were downregulated. Functional enrichment analysis revealed that the apoptosis pathway was activated. The extrinsic apoptosis pathway was activated and the survival factors related pathway was suppressed. Furthermore, gene expressions related to ATP-binding cassette, nuclear factor erythroid 2-related factor, B cell lymphoma-2 family protein, glutathione reductase, glutathione peroxidase, catalase, NADPH2:quinone reductase, and superoxide dismutase were decreased. Conversely, gene expressions related to FAS-associated death domain protein, glutathione *S*-transferase, caspase 6, 8, cytochrome P450 1A1, and 2C8 were increased. These results comprehensively demonstrated the toxicity of the novel algicide TD_49_, and should draw the attention of researchers to the importance of analyzing the potential impact of chemical compounds as algicides to control the proliferation of harmful algae, due to the secondary pollution caused by their application.

## 1. Introduction

In the coastal areas of Northeast Asia, especially Japan, South Korea, and China, scallops are a very important commercial species for marine farming, and account for a high proportion of farmed bivalves [1]. Bivalves, including scallops, are often used as indicators of ocean pollution or as model species in various toxicology studies [2,3] due to their filter-feeding habits and their rapid accumulation of pollutants [4,5]. In recent decades, rapid increases in agriculture and industrial processes, as well as population booms, have substantially increased the frequency, extent, and inducing factors of red tide globally. This increase in red tides may pose a threat to ecology, the aquaculture industry, and human health [6]. Many physiological and ecological investigations have been performed to reduce the severity of losses to coastal fisheries caused by red tides [2,3]. Therefore, developing environmentally friendly algicides has become a major focus of study. In the late 1990s, thiazolidinedione (TD) was developed as an adjunctive therapy for diabetes. TD acts by binding to peroxisome proliferator-activated receptors (PPAR) in the nucleus [6]; it has been shown to be highly effective and selective against harmful algae, with a half maximal inhibitory concentration (IC_50_) in the nanomolar range [7]. The derivative TD 49 (TD_49_) is a newly synthesized algicide that shows high toxicity at low concentrations of 0.1–2 μM against harmful algal species including *Cochlodinium polykrikoides*, *Chattonella marina*, and *Heterosigma akashiwo* [7], as well as very low toxicity to non-harmful algae, even at concentrations of over 100 μM [7]. The new International Organization for Standardization (ISO) standard method, which uses algae, crustaceans, and fish as the three representative species for a marine ecological system, is usually employed for acute toxicity assessments [6]. Beak et al. revealed that 0.6 μM TD_49_ treatment had higher biodiversity indices than the high-concentration treatment (1 μM), which appeared to show a better algicidal effect with phytoplankton biodiversity enhancement [8], and Zooplankton were not affected by TD_49_ concentration of <0.8 μM [9]. Moreover, Kim et al. [6] reported that the LC_50_ of TD_49_ for *Daphnia magna* was 0.68 μM through an acute toxicity assessment. However, in order to comprehensively assess the algicide activity of TD_49_, an acute toxicity assessment on the target species is not sufficient. The low toxicity of the algicide for non-target species should not only be less than its acute toxicity to the target species, but also should have no potential irritation to non-target species, especially economically important aquatic animals. Its potential effects on non-target species at the transcriptional level have not yet been addressed. An overall evaluation of the potential effects of TD_49_ on bivalves is important to effectively establish a method for predicting and evaluating its overall toxicity.

Gene expression profiling is currently widely applied to determine the mechanisms of toxicity induction in chemicals, as well as for predicting their toxicity [1,10,11,12,13]. The Illumina HiSeq 4000 sequencing system is used for extensive genome analysis at a relatively low cost and high output to achieve this [14]. Digital gene expression (DGE) analysis based on this sequencing platform has been applied to a growing number of bivalves, such as bay scallop (*Argopecten irradians*) [13] and *Chlamys farreri* [15] to investigate their responses to environmental stressors. In a previous study, we used next-generation sequencing for gene profiling of the bay scallop, *A. irradians*, in response to a novel algicide, palmitoleic acid (PA). The results identified 5414 genes that were significantly upregulated, as well as 4452 genes that were downregulated [12]. Another previous study by this group also investigated differentially expressed genes (DEGs) and transcript abundance in *A. irradians* gill tissue after exposure to okadaic acid using the Illumina HiSeq 400 deep-sequencing platform, and the results showed that 3204 and 2620 genes were significantly upregulated and downregulated, respectively; these genes played roles in cellular processes, immune system processes, metabolic processes, and catalytic process [13].

The aim of the present study was to perform a comprehensive analysis of the molecular toxicological responses of *A. irradians* upon exposure to TD_49_. The gill is an important respiratory organ in scallops, and acts as a defense barrier owing to its role in the filtration of suspended matter and its high expression of putative immune-related genes [16]. Therefore, the gills were sampled for DGE analysis using the Illumina HiSeq 4000 sequencing system to evaluate whether utilizing TD_49_ as an algicide poses a potential risk to other marine species.

## 2. Materials and Methods

### 2.1. Animal Maintenance and TD_49_ Exposure

Bay scallops, *A. irradians* (average weight: 46.6 ± 3.9 g; average length: 6–7 cm), were purchased from the Zhongcai fisheries wholesale market, Nanjing, China, and maintained in lantern nets suspended in 800 L tanks with filtered and aerated sea water for 2 weeks to acclimatize them to laboratory conditions (temperature: 10 ± 1 °C; salinity: 30% ± 0.1%) before the experiments. Half of the seawater was changed daily before exposure treatment.

For TD_49_ exposure, the bay scallops were randomly divided into two groups: (1) control group and (2) TD_49_-exposed group. TD_49_, which was synthesized according to the methods described by Kim et al. [7], was kindly provided by Professor Hoon Cho, Chosun University (Gwangju, Korea). The TD_49_ was initially dissolved dimethyl sulfoxide (DMSO) (Sigma, Ronkonkoma, NY, USA) to prepare the 50 mM TD_49_ stock solution, and it was ensured that the final concentration of TD_49_ in the TD_49_ treatment group as 0.68 μM without changing the seawater during the 48 h TD_49_ exposure treatment. The scallops in the TD_49_-exposed group were exposed to 0.68 μM TD_49_. The control group was exposed with an equal volume of 0.0125% DMSO in each tank. After exposure to TD_49_ for up to 48 h, the gills from 18 scallops (six scallops with three replicates each) were sampled and stored in 1 mL TRIzol reagent (Invitrogen, Carlsbad, CA, USA) at −80 °C for RNA extraction. Samples from six scallops were pooled from each replicate for RNA extraction.

### 2.2. RNA Preparation

Total RNA was extracted using TRIzol reagent (Invitrogen, Carlsbad, CA, USA) according to the manufacturer’s instructions, and then the purity and rate of degradation of the extracted RNA was measured through 1% agarose gel electrophoresis. Subsequently, a NanoPhotometer spectrophotometer (Implen, Munich, Germany) and a Qubit RNA Assay Kit with a Qubit 2.0 Fluorometer (Life Technologies, Carlsbad, CA, USA) were used to measure RNA purity and contamination, respectively. Finally, the RNA Nano 6000 Assay Kit with the Agilent Bioanalyzer 2100 system (Agilent Technologies, Santa Clara, CA, USA) was used for RNA integrity analysis.

### 2.3. Library Preparation and Illumina Sequencing

Library preparation and Illumina sequencing were performed by the Beijing Genomics Institute (BGI; Beijing, China). Samples of 200 ng DNase I-treated total RNA were purified using oligo-dT beads. In addition, ribosomal and other non-messenger RNAs were removed. Fragmentation was then induced by fragment buffer to remove poly A-containing mRNAs. First-strand and second-strand cDNA were synthesized by using First Strand Master Mix (Illumina, San Diego, CA, USA) with Super Script II (Invitrogen, Carlsbad, CA, USA), and Second Strand Master Mix (Illumina, San Diego, CA, USA), respectively. Then, the overhangs resulting from fragmentation were converted into blunt ends using End Repair Mix. After adding adenylate 3′-End DNA, the ligation reaction was performed using the RNA Index Adapter and Ligation Mix. Next, PCR Primer Cocktail and PCR Master Mix were added to enrich the cDNA fragments. Afterwards, cDNA fragments (260 bp in length) were selected for PCR amplification. The final library was quantified by quantitative PCR (qPCR) with 1 μL of resuspended construct on an Agilent Technologies 2100 Bioanalyzer using Agilent DNA 1000. For cluster generation, the qualified and quantified libraries were first amplified within the flow cell on the cBot instrument (HiSeq 4000 PE Cluster Kit, Illumina, San Diego, CA, USA). For paired-end sequencing, the clustered flow cell was then loaded onto the HiSeq 4000 Sequencer (HiSeq 4000 SBS Kit, Illumina, San Diego, CA, USA) with a recommended read length of 100 bp.

### 2.4. De Novo Transcriptome Assembly

Clean data were obtained using SOAPnuke software by removing the reads with adaptors, low-quality reads, and reads in which unknown bases (N) comprised more than 5% of the reads. Thereafter, the clean reads were assembled using Trinity software to obtain unigenes. The resulting sequences assembled using Trinity were referred to as transcripts. Next, gene family clustering was performed using TIGR Gene Indices clustering tools (TGICL) to obtain the final unigenes. The unigenes were classified into two categories: (1) clusters, labelled with the prefix ‘CL’, followed by the cluster ID, and (2) singletons presented with the prefix ‘unigene’.

### 2.5. Gene Annotation and Analysis

BLAST (version: v2.2.23) was used for functional annotation; the unigenes were aligned to the NCBI nucleotide database (Nt), non-redundant protein sequence database (Nr), SwissProt, the Kyoto Encyclopedia of Genes and Genomes (KEGG), and the Clusters of Orthologous Groups (COG) [17]. Moreover, Blast2GO (version: v2.5.0) and InterProScan5 (version: v5.11-51.0) were used to obtain Gene Ontology (GO) annotations in conjunction with the non-redundant protein sequence database (Nr) annotations and InterPro annotations, respectively [18].

### 2.6. Enrichment Analysis of DEGs

In order to map the high-quality reads to the reference unigene sequences, Bowtie (version: 2.2.5) was applied [19]. Calculation of the unigene expression level was performed using RSEM (version: v1.2.12) [20]. DEGs were detected using PossionDis. PossionDis is based on Poisson distribution, and was performed as described by Audic and Claverie [21]. Furthermore, the threshold values applied were false discovery rate (FDR) < 0.001 and Fold Change ≥ 2.00 to determine the significance of the DEGs.

DEGs were classified according to the standard classification based on the GO annotation results. GO and pathway functional enrichment analyses were also processed using phyper, a function of R. *p*-values were calculated using the hypergeometric was as follows:

$$P=1-\overset{m-1}{\underset{i=0}{\sum }}\frac{\left(\begin{array}{c} M\\ i \end{array}\right)\left(\begin{array}{c} N-M\\ n-i \end{array}\right)}{\left(\begin{array}{c} N\\ n \end{array}\right)}$$

FDR was used for the correction of each *p*-value. FDR < 0.001 was defined as significant enrichment.

### 2.7. Quantitative Real-Time PCR Verification

Ten genes related to detoxification, antioxidant ability, and immune responses were selected for the confirmation of DGE data using qPCR. In an initial study, the efficacy of various reference genes (*GAPDH*, *ef1-α*, and *β-actin*) was evaluated following 48 h exposure to 0.64 μM TD_49_ in scallops. *β-actin* was selected as the housekeeping gene after comparing and ranking the candidate reference genes based on the rankings from three algorithms: GeNorm, NormFinder, and BeestKeeper through RefFinder (https://omictools.com/reffinder-tool) [22]. All specific primers used for qPCR are listed in Supplementary File 1. First-strand cDNA was synthesized with 500 ng total RNA using the PrimeScriptTM RT Reagent Kit (TaKaRa Bio, Shiga, Japan). qPCR was performed at 94 °C for 2 min; followed by 40 cycles at 94 °C for 20 s, 58 °C for 30 s, and 72 °C for 40 s [22], using the QiagenRotor-Gene Q RT-PCR Detection System (Qiagen, Hilden, Germany) in a 12-μL reaction volume (1 μL each primer (10 μM), 6.25 μL SYBR Premix (TaKaRa Bio), 1 μL cDNA (50 ng), and 2.75 μL ultra-pure water). The relative expression of the target genes was calculated according to the method described by Livak and Schmittgen [23]. In all cases, Ct values were determined based on three biological replicates each with two technical replicates.

### 2.8. Statistical Analysis

Statistical analysis was performed using SPSS 19.0 (IBM Corp., Armonk, NY, USA). All data were presented as the mean ± standard deviation (SD). Significant differences were determined by the least significant difference test. Values of *p* < 0.05 were considered statistically significant.

## 3. Results

### 3.1. Analysis of DGE Libraries

**Figure 1:**
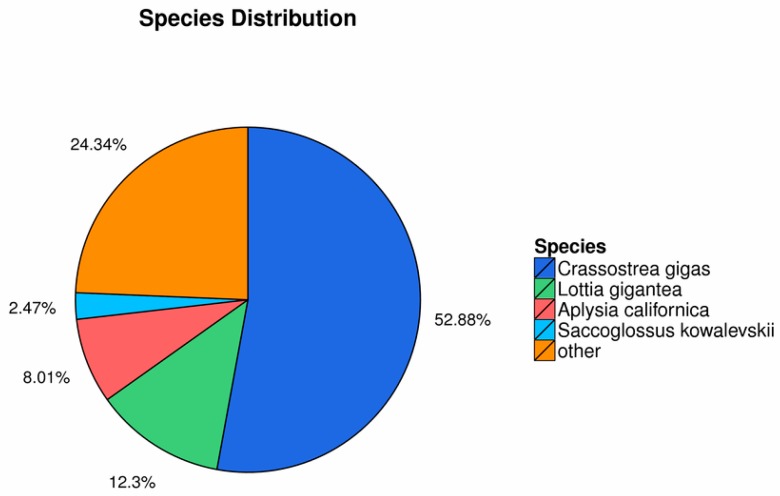
Distribution of annotated species.

After removing reads with adaptors, reads containing poly-N sequences, and low-quality reads from the raw data, we obtained 9.16 Gb total clean bases, with 45.92 Mb clean reads in both the control and TD_49_-treated cDNA libraries. The Q20 and GC percentages of clean reads in the two cDNA libraries were 98.21% and 39.12% for the control cDNA library, and 98.30% and 39.23% for the TD_49_-exposed cDNA library, respectively (Table 1 and Table 2). A total of 78,376 and 71,899 transcripts, with mean sizes of 673 and 632 bp and N50s of 1231 and 1093 bp in the control and TD_49_-exposed groups, were produced using the Trinity tool (Table 2). We thus assumed that the two cDNA libraries were reliable. Finally, transcript sets were further merged with 57,882 unigenes (Table 3). The size distribution of the unigene lengths was as follows: 71.37% (21,073) of the unigenes had a length of 300–1000 bp; 20.88% (6165) had a length of 1000–3000 bp; and 1.77% (524) had lengths of greater than 3000 bp, as shown in Figure 1.

### 3.2. Functional Annotation and Species Distribution

A summary of our functional annotation is shown in Table 4. A total of 28,172 unigenes (accounting for 48.67% of the total unigenes) were annotated in this way. Of these, 25,267 and 9983 unigenes were annotated to the Nr and Nt databases, respectively. There were 19,555 hits via SwissProt, 8729 for COG, 18,931 for KEGG, 4032 for GO, and 19,017 for Interpro (Table 4).

**Figure 2:**
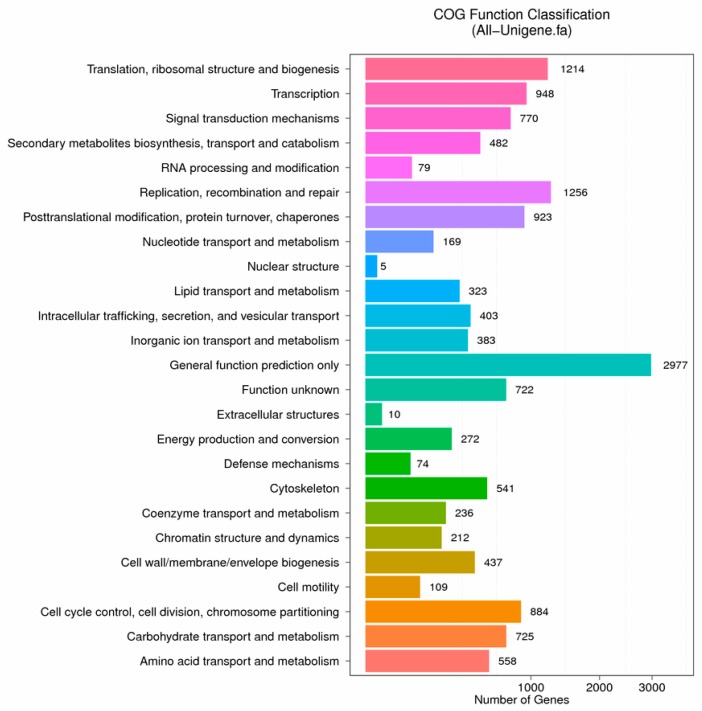
Functional distribution of Clusters of Orthologous Groups (COG) annotation.

The distribution of Nr-annotated species is shown in Figure 1. A total of 14,712 unigenes were aligned to the COG database (Figure 2). The most frequently identified classes were general function (20.24%; 2977); recombinant and repair related (8.54%; 1256); translation, ribosomal structure, and biogenesis (8.25%; 1214); transcription (6.44%; 948); and posttranslational modification, protein turnover, and chaperones related (6.27%; 923).

### 3.3. DEG Analysis

**Figure 3:**
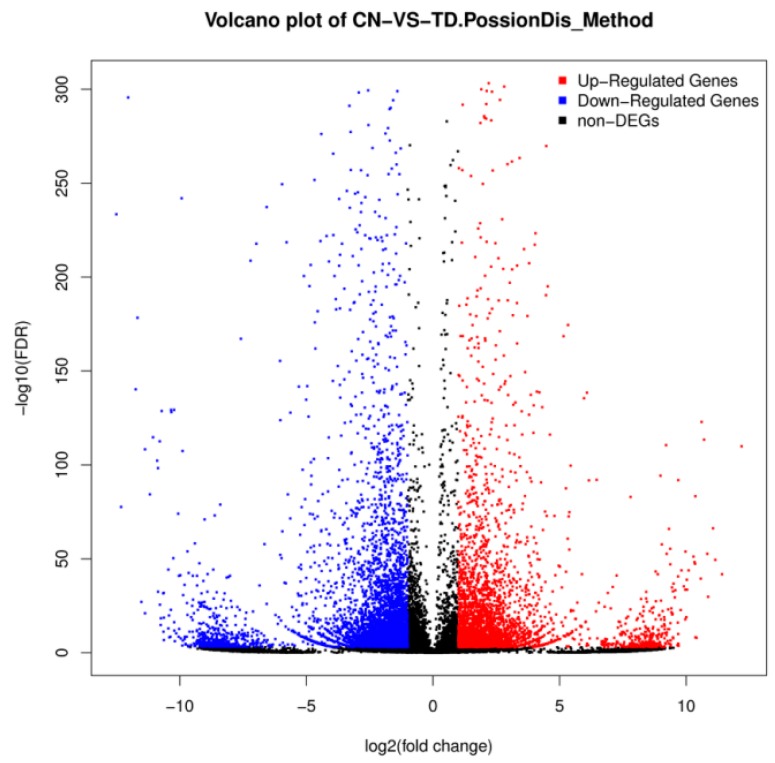
Volcano plot of differentially expressed genes (DEGs) between the control (CN) and Thiazolidinedione (TD)-treated groups.

**Figure 4:**
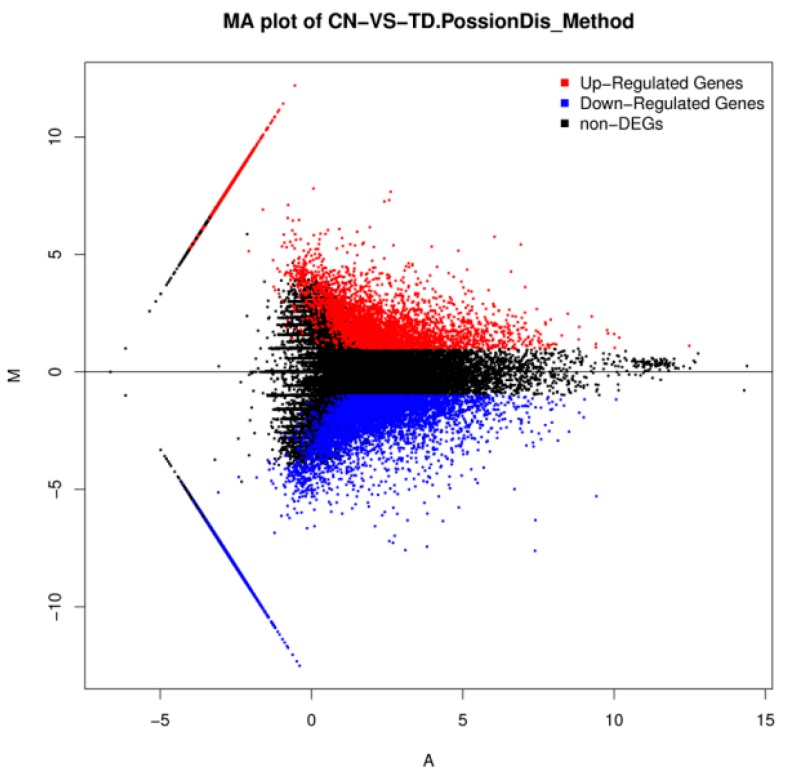
M (log ratio) and A (mean average) (MA) plot of DEGs between the control (CN) and the Thiazolidinedione (TD)-treated group.

Differential expression levels between the control and TD_49_-treated groups are shown in Figure 3 and Figure 4. A total of 8711 unigenes showed differential expression between these two groups (fold change > 2 and FDR ≤ 0.001); among them, 5214 were identified to be upregulated, while 3497 were downregulated (Supplementary File 2).

### 3.4. Enrichment and Pathway Analysis

**Figure 5:**
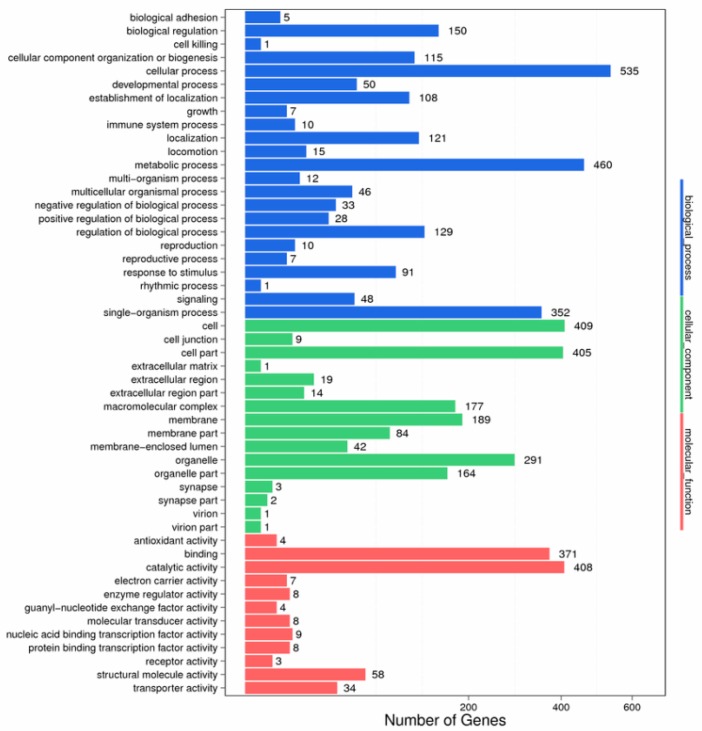
Pathway classification of differentially expressed genes.

Based on GO annotation, a total of 51 functional groups showed substantial enrichment in DEGs compared to the genomic background (Figure 5). Genes related to the terms ‘cellular process’, ‘metabolic process’, ‘cell’, ‘catalytic activity’, and ‘cell part’ were dominant in the TD_49_-exposed scallops. Moreover, the largest percentage of known genes was categorized into biological process and cellular component, followed by molecular function.

**Figure 6:**
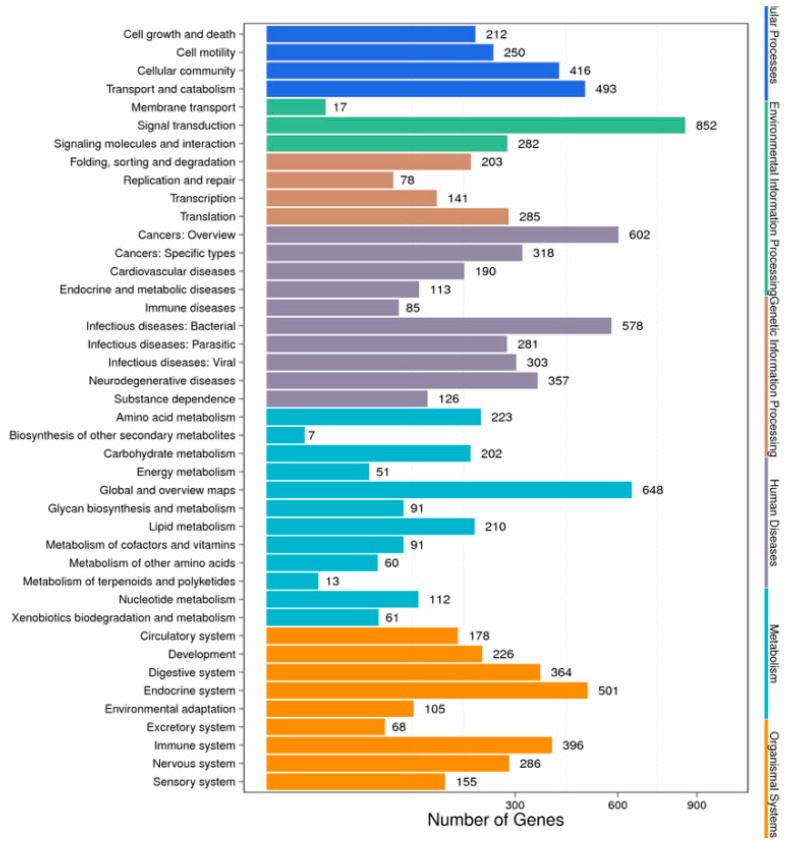
Pathway classification of differentially expressed genes.

**Figure 7:**
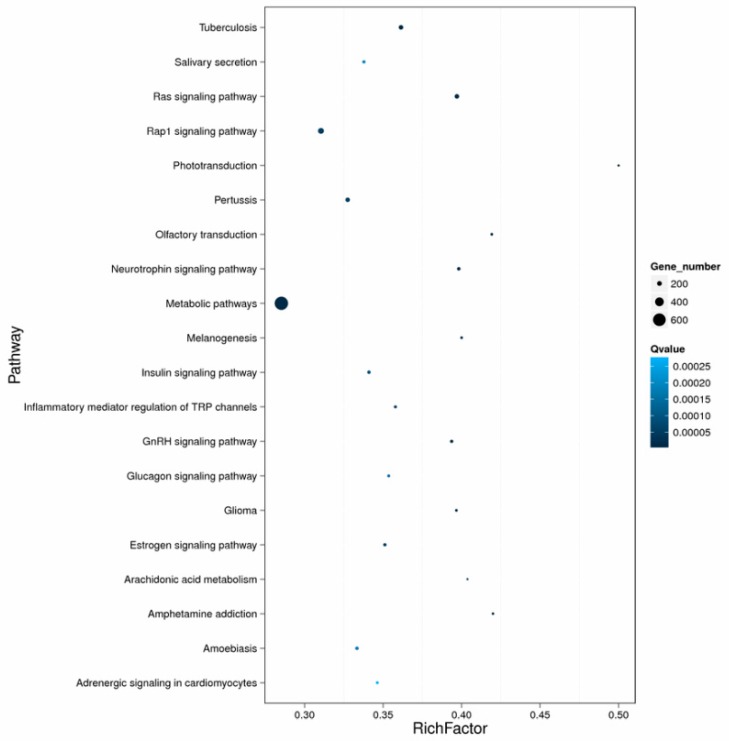
Pathway functional enrichment of differentially expressed genes. Colors indicate q-value (high: white, low: blue), a lower q-value indicates more significantly enriched genes. The size of the dots indicates the degree of differential expression.

**Figure 8:**
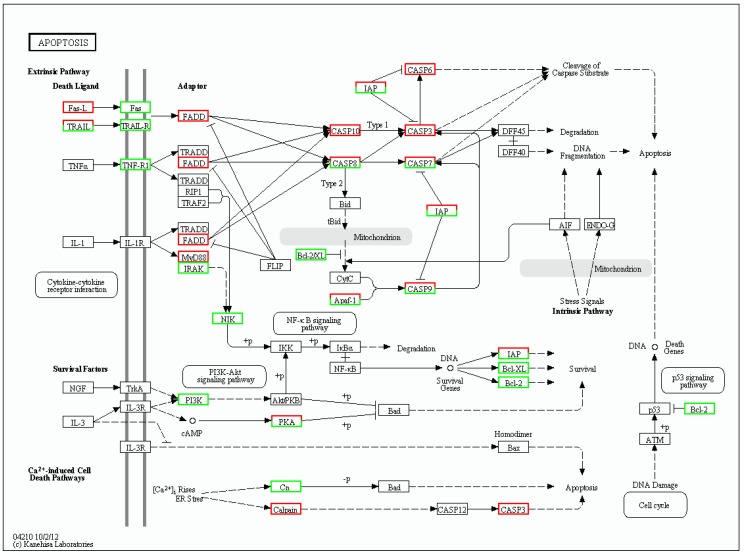
Differentially expressed genes associated with apoptosis pathways. Red, green, and white boxes indicate significantly upregulated, downregulated, and unchanged expression in the transcriptomic profile, respectively.

**Figure 9:**
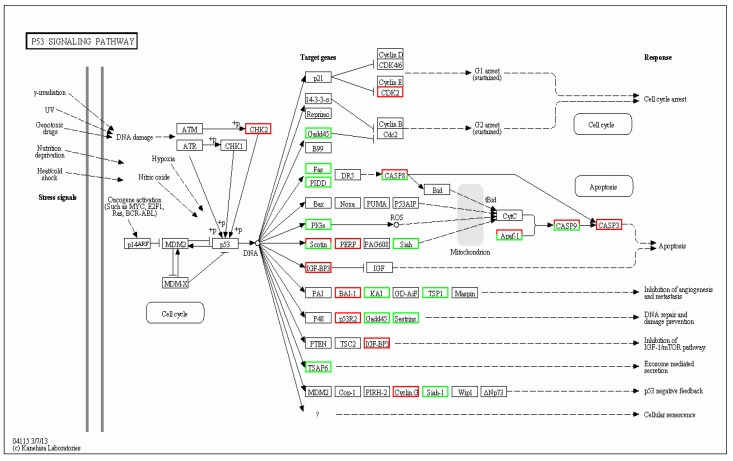
Differentially expressed genes associated with the p53 signaling pathway. Red, green, and white boxes indicate significantly upregulated, downregulated, and unchanged expression in the transcriptomic profile, respectively.

KEGG enrichment analysis of the DEGs showed that they were significantly enriched for signal transduction and metabolic pathways; a total of 3076 DEGs were mapped to 302 pathways, and 105 metabolic pathways were over-represented (corrected *p* < 0.05). Pathway classification and pathway functional enrichment results are shown in Figure 6 and Figure 7, respectively. Among them, 638 genes were annotated to metabolic pathways, 257 to the Rap1 signaling pathway, 208 to the PI3K-Akt signaling pathway, 185 to the Ras signaling pathway, 131 to the mitogen-activated protein kinase (MAPK) signaling pathway, 127 to the cAMP signaling pathway, 86 to the tumor necrosis factor (TNF) signaling pathway, 68 to the nuclear factor-kappa B (NF-κB) signaling pathway, 57 to apoptotic pathways (Figure 8), 50 to the p53 signaling pathway (Figure 9), 44 to the NOD-like receptor signaling pathway, and 42 to the Toll-like receptor (TLR) signaling pathway. The detoxification, apoptosis, and immune-related differentially expressed genes are shown in Table 5.

### 3.5. Genes Related to TD_49_-Induced Stress Response

**Figure 10:**
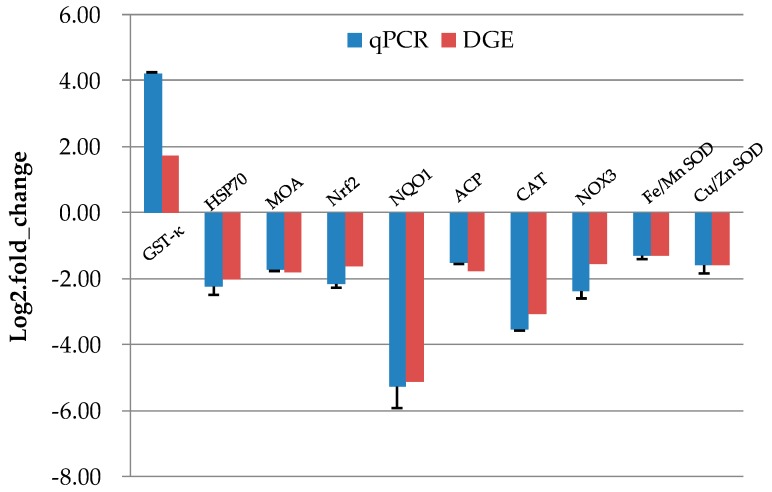
Results of quantitative real time PCR (qPCR) validation (blue) compared to digital gene expression (DGE) analysis results (red). Data represent mean ± SD values (*n* = 3). GST-Kappa: glutathione-*S*-transferase Kappa, HSP70: Heat Shock Protein 70, MOA: monoamine oxidase A, Nrf2: Nuclear factor erythroid 2-related factor 2, NQO1: NAD(P)H:quinine oxidoreductase 1, ACP: acid phosphatase, CAT: catalase, NOX3: NADPH oxidase 3, SOD: superoxide dismutase.

A total of ten transcripts from the DGE libraries were analyzed by qRT-PCR; among them, one was upregulated and nine were downregulated. The detected fold changes from qRT-PCR were compared to those from the DGE analysis results and are shown in Figure 10. The genes exhibited a consistent trend for both qRT-PCR analysis and DGE results. The correlation coefficient between the DGE and qRT-PCR results was 0.96 (*p* < 0.001).

## 4. Discussion

For a comprehensive environmental risk assessment, exposure assessment, dose-response assessment, hazard identification, and risk characterization are widely used as standard protocols. TD_49_ is a newly synthesized algicide that shows high toxicity to harmful algae at low concentrations of 0.1–2 μM, but shows very low toxicity to non-harmful algae, even at concentrations of >100 μM [7]. However, in the application of TD_49_ as algicide, TD_49_ exposure may also cause potential irritation to non-target species, especially for economically important aquatic animals. The current study revealed the overall responses to TD_49_ exposure by bivalves at the transcript level using transcriptomics analysis, and effectively helped to establish a standard method for predicting and evaluating the toxicity of a novel algicide in bivalves.

Apoptosis is a process of programmed cell death that is characterized by the activation of caspases (Cas), followed by the systematic breakdown of dying cells into vulnerable phagocytized apoptosomes [24]. Members of the antiapoptotic B cell lymphoma-2 family (Bcl-2) including Bcl-XL protects cells from a series of apoptotic factors and are essential for cell survival [25]. In the current study, we found that the expression of Bcl-2 and Bcl-XL was decreased, suggesting that TD_49_ exposure inhibited the expression of survival factors. In addition, inhibitors of apoptosis (IAP) family proteins, which are characterized by a novel ~70-amino acid domain known as the baculoviral IAP repeat (BIR), are further important negative regulators of apoptosis [26,27]. In the present study, the expression of baculoviral IAP repeat-containing protein (BIRC)-related genes including *BIRC 2*, *BIRC 3*, *BIRC 6*, *BIRC 7A*, and *BIRC 7B* was downregulated, indicating the potential activation of downstream caspase. Meanwhile, elevated *Cas3*, *Cas6*, *Cas10* and FAS-associated death domain protein (*FADD*) gene expression levels in the gills of TD_49_-exposed bay scallops revealed that TD_49_ induces FADD-dependent apoptosis in the gills of bay scallops, thereby inducing apoptosis primarily through the activation of Cas10 and its downstream signaling pathway [28]. Moreover, TD_49_ exposure also activated apical caspases including Cas8 and Cas9, which then activated the downstream effector Cas3, resulting in cell death.

ATP-binding cassette (ABC) transporters are major transmembrane proteins, and comprise the largest transporter gene family. They play a role in transporting a series of substrates including proteins, sugars, amino acids, peptides, and metal ions, as well as numerous hydrophobic compounds and metabolites across biological membranes [29]. In addition, in aquatic species, they play a part in multiple xenobiotic resistance phenotypes by exporting xenobiotic substances out of the cells or by facilitating the sequestration of toxins within specialized cells or organelles, effectively segregating them from vulnerable protein and DNA targets [12,29]. ABCB1 has been reported to protect cells from toxic factors due to its broad substrate specificity; ABCG2 is similarly involved the transport and efflux of drugs or other toxic compounds [30,31]. ABCC1 was identified as a multidrug resistance gene and has been demonstrated to transport glutathione conjugates originating from many toxic compounds [30]. Therefore, the decreased expression of *ABCB1*, *ABCC1*, and *ABCG2* in the gills of bay scallops exposed to TD_49_ may indicate a reduced ability to resist a wide range of xenotoxins or endogenous stimulants. Previous reports have found that ABCA, ABCG, and ABCD classes act as gatekeepers for the cellular and body homeostasis of sterols, and may exert related functions for the cellular homeostasis of phospholipids and cholesterol and fatty acid metabolism in vertebrates [30]. Hence, the inhibition of *ABCA3*, *ABCA5*, and *ABCD3* expression induced metabolic disequilibrium in TD_49_-exposed bay scallops. Cytochrome P450s (CYP) make up a major family of enzymes which act on many endogenous substrates including fatty acids, eicosanoids, bile acids, sterols and steroids, retinoids, vitamin D3 derivatives, and uroporphyrinogens [32]. NADPH oxidase (NOX), including NOX3, is found in the cytoplasmic membrane of phagocytic cells and can generate reactive oxygen species (ROS) to remove pathogenic invaders [33]. The current results showed that *NOX3* mRNA expression was suppressed following TD_49_ exposure, and it was speculated that the elimination of TD_49_ was not due to NOX3-mediated ROS production. Moreover, many cytochrome P450 enzymes can metabolize various exogenous compounds including natural plant products, drugs, environmental chemicals, and other contaminants, subsequently resulting in the successful detoxication of the irritant [32]. Among them, CYP 1A1 and CYP 2C8 were identified to be involved in the metabolism of xenobiotics [34]. In our present investigation, the expression of *CYP 1A1* and *CYP 2C8* was upregulated, indicating that these proteins activated in response to TD_49_ invasion. In addition, GSTs represent a major family of detoxification enzymes [35]. The elevation of *GST ζ1, GST 1, GST σ3, GST A*, and *GST κ1* mRNA in the gills of bay scallops after TD_49_ exposure compared to control implied that the clearing of TD_49_ was mainly carried out by GST.

The antioxidant defense system functions in the elimination of free radicals and is vital for the response to oxidative stresses and damage. Antioxidant enzymes including catalase (CAT), superoxide dismutase (SOD), glutathione peroxidase (GPx), reductase (GR), and NAD(P)H:quinine oxidoreductase 1 (NQO1) play important roles in detoxification. SOD removes superoxide anions from the cytoplasm by catalyzing the dismutation of two superoxide radicals to hydrogen peroxide (H_2_O_2_) and oxygen (O_2_) [36]. Thereafter, H_2_O_2_ is catalyzed by CAT to produce H_2_O and O_2_ [37]. GPx and GR are also important antioxidative enzymes; when H_2_O toxicity is neutralized by different GPx members, GSH is converted to oxidized glutathione (GSSG), and subsequently reduced by GR, thus maintaining the GSH/GSSG ratio [38]. In the present study, the expression of *SOD, CAT, GPx*, and *GR* were inhibited, suggesting that the antioxidant capacity of TD_49_-exposed bay scallops was strongly inhibited. NQO1 is widely known to have multiple protective functions; it can catalyze the reduction of nitroaromatics, quinoneimines, quinones, and azo dyes compounds, as well as protect cells from redox cycling and oxidative stress [12,39]. The present results showed that the expression of *NQO1* mRNA in bay scallops decreased after TD_49_ exposure, indicating that TD_49_ acted as an inhibitor of NQO1. Therefore, it can be deduced that TD_49_ exposure may diminish the protective ability of cells against oxidative damage and toxic effects, including those caused by quinones. Nrf2 binds to the antioxidant-response element (ARE) and regulates ARE-mediated antioxidant enzyme gene expression in response to a variety of stimuli, including metals, xenobiotics, antioxidants, and UV irradiation [40]. Previous studies demonstrated that Nrf2 can regulate ARE-mediated *NQO1* gene expression, and is subsequently involved in the transcriptional activation of other ARE-mediated genes including haem oxygenase 1 (*HO-1*), the *GST Ya* subunit, and γ-glutamylcysteine synthetase (*γ-GCS*), proteasomes, and drug transporters [40]. In our current investigation, *Nrf2* expression was majorly downregulated, consistent with a previous study showing that PA exposure substantially inhibited the expression of *Nrf2* in bay scallops [12]. Hence, we speculated that TD_49_ suppressed the expression of *Nrf2*, thereby decreasing the expression of downstream antioxidant-related genes including *NQO1*, *SOD*, *CAT*, *GPx*, and *GR* [41].

In invertebrates, the innate immune system mainly functions in the prevention of pathogenic invasion, as they lack an acquired immunity. Immune recognition, which discriminates self and non-self, plays a crucial part in initiating the immune response [11]. Immune responses begin when soluble, specialized, or cell-bound pattern recognition receptors (PRRs) recognize pathogen-associated molecular patterns [11,31]. To date, seven distinct PRRs have been identified in scallops, including fibrinogen C domain-containing protein (FIBCD) and C-type lectins (CLEC). In the present study, *TLR*, *CLEC 4M*, *CLEC 4F*, and *FIBCD 1* mRNA expression was majorly inhibited after exposure to TD_49_, indicating that TD_49_ exposure might suppress the ability of scallops to recognize and clear non-self molecules such as pathogenic bacteria and xenobiotics. The related expression of acid phosphatase (*ACP*), which is a typical lysosomal enzyme sensitive to environmental stresses [42,43], was reduced in the gills of bay scallops exposed to TD_49_. This is consistent with a previous study, which reported a decrease in ACP expression in the gills of bay scallops after PA exposure for up to 48 h [12].

In conclusion, we have comprehensively revealed the transcriptional complexity of the physical responses of bay scallop gills to TD_49_ exposure, many of which are related to the pathways controlling apoptosis, detoxification, immune response, and antioxidant processes against TD_49_. Moreover, the current results demonstrated the potential toxicity of the algicide TD_49_, which might act as an environmental endocrine disruptor for non-target marine species, especially economically important bivalves, since TD_49_ exposure appeared to activate the apoptosis pathway. Furthermore, the results of this study not only revealed the potential risk of the mainstream application of TD_49_ as an algicide strategy, but should also draw the attention of researchers to the importance of analyzing the potential impact of chemical compounds as algicides to control the proliferation of harmful algae, due to the secondary pollution caused by their application.

## Figures and Tables

**Table 1:** Summary of sequencing reads after filtering.

Sample	Total Raw Reads (Mb)	Total Clean Reads (Mb)	Total Clean Bases (Gb)	Clean Reads Q20 (%)	Clean Reads Q30 (%)	Clean Reads Ratio (%)
CN	45.92	45.76	4.58	98.21	95.30	99.65
TD	45.92	45.85	4.58	98.30	95.52	99.84

Q20: the rate of bases with a quality of greater than 20, CN: control, TD: Thiazolidinedione 49-exposed.

**Table 2:** Quality metrics of transcripts.

Sample	Total Number	Total Length	Mean Length	N50	N70	N90	GC (%)
CN	78,376	52,817,747	673	1231	568	252	39.12
TD	71,899	45,492,111	632	1093	518	245	39.23

N50: a weighted median statistic showing that ≥50% of the total length is contained in the transcripts, GC (%): the percentage of G and C bases in all transcripts, CN: control, TD: Thiazolidinedione 49-exposed.

**Table 3:** Quality metrics of unigenes.

Sample	Total Number	Total Length	Mean Length (bp)	N50	N70	N90	GC (%)
CN	51,394	40,985,988	797	1410	702	302	39.48
TD	48,654	36,217,952	744	1266	637	288	39.54
All-unigene	57,882	50,329,144	869	1594	827	321	39.36

N50: a weighted median statistic indicating that ≥50% of the total length is contained in the unigenes, GC (%): the percentage of G and C bases in all unigenes, CN: control, TD: Thiazolidinedione 49-exposed.

**Table 4:** Summary of functional annotation of unigenes.

Values	Total	Nr- Annotated	Nt- Annotated	SwissProt- Annotated	KEGG- Annotated	COG- Annotated	Interpro- Annotated	GO- Annotated	Overall
Number	57,882	25,267	9983	19,555	18,931	8729	19,017	4032	28,172
Percentage	100%	43.65%	17.25%	33.78%	32.71%	15.08%	32.85%	6.97%	48.67%

**Table 5:** Detoxification, apoptosis, and immune-related differentially expressed genes in gills of bay scallop exposed to Thiazolidinedione 49 (TD_49_) for up to 48 h.

Description	Transcript	Log2 Fold Change (RNAseq)	Regulation
Downregulated			
Immune system	CLEC 4M	−1.62	Down
	CLEC 4F	−2.69	Down
	FIBCD 1	−2.00	Down
	ACP 5	−2.94	Down
	ACP 6	−2.40	Down
	PAPL	−1.27	Down
	HSP70	−2.00	Down
Apoptosis	Bcl-2	−1.90	Down
	BIRC 2	−2.51	Down
	BIRC 3	−4.97	Down
	BIRC 6	−4.25	Down
	BIRC 7A	−1.33	Down
	BIRC 7B	−1.61	Down
Transmembrane proteins	ABC A3	−5.01	Down
	ABC A5	−2.80	Down
	ABC B1	−2.11	Down
	ABC B10	−4.27	Down
	ABC C1	−1.16	Down
	ABC D3	−1.32	Down
	ABC G2	−1.95	Down
Antioxidant system	NQO1	−5.10	Down
	CAT	−3.06	Down
	Mn SOD	−1.30	Down
	Cu/Zn SOD	−1.56	Down
	GR	−5.08	Down
	GPx	−2.55	Down
	Nrf2	−1.61	Down
Metabolism of xenobiotics	CREBP 3	−1.39	Down
	MOA	−1.79	Down
	NOX3	−1.55	Down
	GSTω	−11.18	Down
Upregulated			
Apoptosis	Cas3	2.29	Up
	Cas6	2.79	Up
	FADD	1.68	Up
Immune system	TLR 2	4.18	Up
	TRAF 6	1.17	Up
Metabolism of xenobiotics	CYP 1A1	2.45	Up
	CYP 2C8	8.52	Up
	GST *ζ*1	8.40	Up
	GST 1	3.08	Up
	GST*σ*3	3.20	Up
	GST A	3.41	Up
	GST*κ*1	1.74	Up

CLEC: C-type lectin domain family, FIBCD: Fibrinogen C domain-containing protein, TLR: Toll-like receptor, ACP: Lysosomal acid phosphatase, PAPL: Iron/zinc purple acid phosphatase, ABC: ATP-binding cassette sub-family, CAT: Catalase, GR: Glutathione reductase, GST: Glutathione *S*-transferase, GPx: glutathione peroxidase, Bcl-2: B cell lymphoma-2 family protein, CREBP: Cyclic AMP-responsive element-binding protein, CYP: Cytochrome P450, Nrf2: Nuclear factor erythroid 2-related factor 2, NQO1: NAD(P)H:quinine oxidoreductase 1, NOX3: NADPH oxidase 3, BIRC: Baculoviral IAP repeat-containing protein, FADD: FAS-associated death domain protein, Cas: caspase, TRAF: tumor necrosis factor receptor-associated factor, MOA: flavin-containing monoamine oxidase A, HSP70: Heat shock protein 70, SOD: superoxide dismutase.

## Supplementary Materials

The following are available online at https://www.mdpi.com/2218-273X/9/8/310/s1. Supplementary File 1: The differential expression unigenes between the control and the TD_49_-treated groups. The accession number for our Raw dataset was submitted to GEO database under the accession number GSE133024.
